# Linking Digital Capacity to Innovation Performance: the Mediating Role of Absorptive Capacity

**DOI:** 10.1007/s13132-022-01092-w

**Published:** 2022-10-24

**Authors:** Ioanna Kastelli, Petros Dimas, Dimitrios Stamopoulos, Aggelos Tsakanikas

**Affiliations:** 1 Laboratory of Industrial and Energy Economics, National Technical University of Athens, Zografou Campus, 9 Iroon Polytechniou str, 15780 Zografou, Greece; 2 Hellenic Open University, Patras, Greece; 3 Laboratory of Industrial and Energy Economics, National Technical University of Athens, Zografou, Greece; 4 Laboratory of Industrial and Energy Economics , National Technical University of Athens, Zografou, Greece; 5 Laboratory of Industrial and Energy Economics, National Technical University of Athens, Zografou, Greece

**Keywords:** Innovation, Digital capacity, Absorptive capacity, Digital transformation, Greece, D22, O31, O32, O33

## Abstract

Digital technologies are considered as factors that accelerate the pace of innovation and increase the firm’s innovation performance. However, few studies have investigated whether this claim is conditioned by other elements that contribute to innovation. Furthermore, firms increasingly rely on external knowledge sources to expand their internal knowledge base for the development of innovations. In this context, absorptive capacity can be considered as an essential organizational capability to embrace adoption of digital technologies and enhance their positive effect on innovation performance. This paper builds on this discussion and studies the contribution of digital capacity on innovation performance, proposing the mediating role of absorptive capacity in the context of the digital transformation. It uses evidence from an extensive Greek survey in 1014 manufacturing firms and analyzes the complex relationships underlying the role of digital transformation to innovation. The contribution of the paper is two-fold: (i) it provides a deeper insight into the underlying mechanisms through which firms can leverage their digital capacity to accelerate innovation, and (ii) it highlights the important mediating role of absorptive capacity in enhancing the positive effects of digitalization indicating that digital capacity is not an unquestionable asset for innovation performance. Accordingly, our results show a positive direct contribution of digital capacity to innovation performance, which is enhanced in the presence of absorptive capacity as a mediator. In fact, the indirect effect of digital capacity to innovation performance through absorptive capacity is stronger. These findings present important policy implications, as there is need for improvement in other innovation-related aspects of the business ecosystem to efficiently address the challenge of digital transformation, such as R&D efforts, training, interaction among actors, and building of communities of practice.

## Introduction

As the world is heading towards the first quarter of the twenty-first century, the rapid technological advancements of Industry 4.0 (I4.0) have urged organizations and businesses to adopt new digital-based systems and integrate knowledge-intensive elements to achieve their desired innovation performance and better position themselves in the ever-evolving landscape of the new digital world (Brynjolfsson & McAfee, 2014; Mubarak & Petraite, 2020; Müller et al., 2018; Scuotto et al., 2017). The development and diffusion of general-purpose technologies (GTPs), such as the information and communication technologies (ICTs), share specific characteristics of pervasiveness, dynamism, and complementarity (Bresnahan, 2010; Martinelli et al., 2021). I4.0 has brought forward a large set of digital technologies that meet the criteria of GTPs, such as the internet of things (IoT), big data, and artificial intelligence that follow different trajectories across industries and locations and can act both as enabling factors and proxies for the digital transformation (Alejandro G. ). The adoption of these new digital technologies was significantly amplified under the unprecedented pressures derived from the COVID-19 pandemic, as they provided a much-needed set of tools that enhance enterprise agility as a response mechanism of adaption to unprecedent market challenges (Overby et al., 2006; Wei et al., 2017).

In this line, many studies investigate the determinant factors of moving from adopting an I4.0 technology to innovation. In fact, in the complex process of innovation development, other significant factors come into play that relate with each firm’s dynamic capabilities and economic competencies. Such elements are prerequisites for I4.0 technologies and relevant digital skills to facilitate and advance a firm’s innovation performance (Ciarli et al., 2021; Scuotto et al., 2017, 2022). In this context, a few attempts questioned the effect of digital technologies on innovation as they put forward the issues of reduced social interaction, loss of the tacit aspects of the knowledge flows, and standardization of knowledge that is easily replicated by competitors (Lember et al., 2019; Palacios-Marqués et al., 2016; Usai et al., 2021).

In light of the above, the aim of this paper is to study the impact of digital capacity on innovation performance, using evidence mainly from the Greek manufacturing sector. We define digital capacity as a digital transformation driver, namely, a factor that enables the implementation of transformation. We relate it to the potential of firms to adopt and use digital technologies, digitalize their offerings, and create new value from digitally driven business models by shifting for example from product-centric to digitally based servitization activities; to the restructuring of business functions such as for example purchase and supply management or new product development; to the employment of high-skilled workforce (Ciarli et al., 2021; Paiola & Gebauer, 2020; Scuotto et al., 2022; Siachou et al., 2021; Wehrle et al., 2021).

Furthermore, the paper explores the role of absorptive capacity as a mediating factor in the creation of value innovation. Building on this main research question, the contribution of this paper is two-fold. First, to identify the role of absorptive capacity as an antecedent of the digital transformation of enterprises and, second, to contribute to the better understanding of the underlying mechanisms through which enterprises can leverage their digital capacity to accelerate innovation.

The empirical research is based on a Greek survey to industrial firms and investigates the contribution of digital capacity and absorptive capacity (measured as R&D intensity, training efforts, and innovation co-operations) to the innovation output (product, process, and marketing/distribution) of a diverse sample of 1014 Greek enterprises from different sectors and regions. Greece is an interesting case as while considered as a *moderator* in the latest European Innovation Scoreboard rankings, it is consistently ranked at the bottom of relevant digitalization indicators and reports,^1^ raising interest regarding the extent to which recent technological advancements and digital technologies can be identified as significant factors that directly affect the innovation performance of its enterprises.

Overall, our findings support to some extent that emerging digital technologies contribute to innovation performance. However, our analysis goes deeper into the mechanisms through which digitalization can play a positive role for supporting and enhancing innovation and accordingly strengthen the firm’s competitive advantage. Our results highlight the strong positive direct contribution of absorptive capacity to innovation performance and support the important mediating role of absorptive capacity in enhancing the positive effects of digitalization. It is then highlighted that digitalization is not unquestionable in fostering innovation performance.

The remainder of this paper is structured as follows. In the second section, we discuss the theoretical implications that provide the background for the formulation of our research hypotheses. In the third section, we present the empirical research, including the methodology, the formulation of the statistical constructs (variables), and the estimation technique. In the fourth section, we present the results obtained using the PLS-SEM method as the most appropriate for the type of relationships we test. The fifth section presents the discussion on the empirical results and the sixth the conclusions. A final section provides some limitations of the research and future research steps.

## Conceptual Framework

### The Role and Relevance of Digital Capacity in Accelerating Innovation

It is widely acknowledged that emerging digital technologies improve/accelerate innovation through organizational transformation (Boeker et al., 2021; Carayannis et al., 2006; Verstegen et al., 2019). Internet expansion, e-commerce development, and use of emerging technologies such as IoT, artificial intelligence, and blockchain increase opportunities for firms to enter global markets and reach customers all over the world. The firm faces a greater range of potential options for action, seeking investment profits.

There are different perspectives in the literature when studying the digital transformation at the firm level. An attempt to synthesize them would imply that it is about adopting new technologies, changing organizational structures, using social media and e-platforms to interact with users, opening new channels of sales, and/or establishing new ways of doing business (Carayannis et al., 2006; Scuotto et al., 2017, 2022; Tekic & Koroteev, 2019). The adoption of digital technologies can radically change the knowledge generation process of firms as it relates to the successful integration and exploitation of advanced ICTs in the firm’s functions (Brynjolfsson & McAfee, 2014) and provides a softer source of innovation (Tether, 2005) which relates to changes in the way of doing business, the interaction with other actors of the business environment, and the overall business model itself (Müller et al., 2018). Furthermore, other dimensions of the phenomenon relate to direct and indirect gains in terms of production efficiency, overall performance (e.g., returns on sales), and product quality (Blichfeldt & Faullant, 2021; Dalenogare et al., 2018; Kagermann, 2015; Porter & Heppelmann, 2014; Yunis et al., 2018).

The digitalization process of firms includes the convergence of several types of I4.0 emerging technologies which results into novel cyber-physical and intelligent systems that can create or add value to several industrial activities (Frank et al., 2019a; Muller et al., 2018; Liao et al., 2017). In fact, as Reischauer (2018) states, I4.0 should be treated as a policy-driven innovation discourse and a communicative action that aims to mobilize different actors that include the business ecosystem, academia, and state, to actively collaborate to produce innovation. In this line, several studies have investigated the link between the adoption of I4.0 technologies and the production of innovation under various settings and for different types of innovation such as product/service innovation (Blichfeldt & Faullant, 2021; Sarbu, 2021) and business model innovation (Frank et al., 2019b; Muller et al., 2018), unveiling a positive link between the two.

However, is this relationship of digital transformation with innovation performance so straightforward and unquestionable? A recent study by Usai et al. (2021) presented some intriguing findings. More specifically, they challenged the established status-quo by providing empirical evidence that digital technologies have a very low impact on innovation and the actual innovation performance predictor is the firm’s R&D expenses. For the conceptual consolidation of their findings, they turned to Yoo et al. (2012) and argued that the generative and combinatory properties of I4.0 technologies are in fact inhibiting factors for innovation performance, as they entail reusable and imitable knowledge. Instead, they put forward the argument that the focus for the stimulation of innovation performance should reside with the development of each firm’s unique knowledge sources. Digital technologies might have little or no direct impact on innovation performance because they interplay with other elements and their effect is conditioned by what the firm uniquely knows and creates either internally or in collaboration with others (Usai et al., 2021). However, the digital transformation is not only about adopting digital technologies. Another significant dimension goes beyond adoption and relates with the effective use and exploitation of digital technologies and the development of digital capabilities towards the production of innovations (Zammuto et al., 2007). In this process, employees are required to develop a set of *metaskills* that enable them to adapt and/or expand their existing skillset in accordance with the evolution and ever-expanding introduction of new digital technologies (Ciarli et al., 2021).

For firms to accomplish their digital transformation, they need to develop their digital capacity which encompasses efforts to integrate and actively utilize digital technologies, transform their functions, and develop their human resources to adopt and take advantage of digital technologies. In general, digital capacity could be related to two dimensions. First, a supply-side dimension which refers to the discovery, production, and supply of digital technologies, which is more explorative in nature. This side is relevant for firms that generate these technologies. However, as digital technologies are horizontal and can be shared across various activities and knowledge bases, a second dimension relating to the demand-side reveals great importance and refers to the adoption, integration, use, and implementation of digital technologies and is more exploitative in nature. It is relevant for firms that adopt digital technologies to efficiently transform their business processes and seize their impact on their business models and operational activities.

In this study, we focus on the second dimension of digital capacity as a digital transformation driver, namely, a factor that enables the implementation of transformation. It relates to the potential of firms to adopt and use digital technologies, digitalize their offerings, and create new value from digitally driven business models by shifting for example from product-centric to digitally based servitization activities; to the restructuring of business functions such as for example purchase and supply management or new product development; to the employment of high-skilled workforce (Ciarli et al., 2021; Paiola & Gebauer, 2020; Scuotto et al., 2022; Siachou et al., 2021; Wehrle et al., 2021). To further elucidate each firm’s digital capacity, we can distinguish between two groups of digital technologies, based on their relation to the innovation process. The first group comprises physical technologies that relate to the production process and the development of innovation per se, while the second group includes data-based, intangible technologies that cover the overall business functions horizontally and support the innovation process indirectly. Hence, we can refer to the first group as production-based technologies and to the second group as management-based technologies.

Combining the different perspectives from our discussion above, we argue that the impact of digital technologies should not be unquestionable but should rather be studied in conjunction with other innovation capabilities. Accordingly, we formulate our first research hypothesis:

- H1: Digital capacity increases innovation performance of firms.

Of course, as stated above, mere adoption of digital technologies does not necessarily imply innovation, as the effective use of the said technologies is a critical element in the digitalization process. These technologies are characterized by strong complementarities as their adoption relates to incorporation of complex systems of interrelated tangible and intangible parts and requires technical and managerial skills, organizational capital, innovation, and financing capacity to generate benefits (OECD, 2019). Building on this implication, this study puts forward the argument that innovation performance depends not only on digital capacity directly, but also more importantly on the firm’s existing knowledge base and its own capabilities to acquire, assimilate, and effectively use digital technologies for the creation of new value. In this line, we suggest that digital transformation mainly occurs in the presence of absorptive capacity and investigate their conceptual linkage in the following section.

### Absorptive Capacity as a Mediator Between Digital Capacity and Innovation Performance

Economic and management literature puts emphasis on interaction as a mechanism of knowledge creation and on the importance of both internal and external knowledge sources to develop innovations (Chesbrough, 2003a, b; Nonaka & Takeuchi, 1995; Von Hippel, 1998). Cohen and Levinthal (1989, 1990) have studied the ability to exploit external knowledge as being critical to innovative performance, introducing the concept of absorptive capacity at the level of the firm. Thus, absorptive capacity refers not only to the acquisition and assimilation of information by an organization but also to the organization’s ability to exploit it. Its main attributes are that it is path dependent and cumulative (Cohen & Levinthal, 1990). According to both authors, absorptive capacity relies on two important elements: the existing knowledge base and the intensity of efforts made for the development of technological capabilities. The existing knowledge base increases the ability to search, recognize, and represent a problem as well as assimilate and use new knowledge for problem solving. The intensity of effort or commitment in problem solving refers to the amount of energy that organizational members devote to solve problems (Kim, 1999). The capability of a firm to absorb knowledge and information from external sources is one of the pillars in the process of transformation of knowledge and information into new knowledge and its conversion into new value.

Since Cohen and Levinthal’s seminal work, there have been a lot of theoretical and empirical research on that issue. Zahra and George (2002) built upon the work of Eisenhardt and Martin (2000) referring to dynamic capabilities and defined AC as a set of organizational routines and processes by which firms acquire, assimilate, transform, and exploit knowledge to produce a dynamic organizational capability. Other studies have elaborated on the definition of AC and many theoretical considerations as well as empirical studies have converged to the conclusion that absorptive capacity is a complex construct consisting of a set of discrete organizational routines and processes. They also present empirical evidence for the role of prior knowledge and intensity of efforts or commitment in problem solving and of interaction with external sources of knowledge in improving the effectiveness of learning and innovative processes (Ávila, 2021; Caloghirou et al., 2004; Carayannis et al., 2006; Eisenhardt & Martin, 2000; Jiménez-Barrionuevo et al., 2011; Kastelli et al., 2004; Lane & Lubatkin, 1998; Lane et al., 2001; Larrañeta et al., 2017; Laursen & Salter, 2006; Mowery et al., 1996; Proeger, 2020; Zahra & George, 2002).

There is a further issue to be discussed on AC, which relates to the measurement of a complex construct. Our definition of AC as a set of organizational routines and processes by which firms acquire, assimilate, transform, and exploit knowledge to produce a dynamic organizational capability points to the consideration of mechanisms related to the intensity of efforts made for the development of firms’ internal capabilities and for accessing knowledge from external sources (Caloghirou et al., 2004). Several studies measure absorptive capacity using selected proxies (e.g., Cohen & Levinthal, 1990; D’Souza & Kulkarni, 2015), or through the construction of latent variables (e.g., Jansen et al., 2005; Revilla et al., 2013; Szulanski, 1996). In this research, we operationalize AC by looking at different dimensions of the construct. We are interested not only in internal processes of the firm such as R&D activity but also in organizational practices supporting knowledge diffusion within the firm and in interactions of the firm with other actors. We consider that what constitutes absorptive capacity is a system of elements that relate to prior knowledge and experience, learning, and creative efforts to develop the firm’s knowledge base (such as R&D and training), knowledge diffusion efforts within the firm, and interactive efforts to take advantage of external sources of knowledge. Prior knowledge and experience are embedded in the firm’s human capital and organizational routines. Technology adoption requires skilled employees and established practices for the development of firm’s human capital to understand and integrate new knowledge related to digital technologies and enable transformation for developing new products, processes, or other forms of innovation. Investment in R&D represents an exploration and discovery mechanism that may enable the exploitation of digital technologies through innovation activity. Collaboration with other organizations extends firm’s capability to negotiate, communicate, and manage external relationships and establishes a culture to cooperate and share knowledge and experience (Anand & Khanna, 2000; Barajas & Huergo, 2010; Gulati et al., 2000).

This discussion gains more legitimacy in the context of open innovation strategy as the innovation process is increasingly relying on purposive inflows and outflows of knowledge and is based on both internal and external knowledge sources such as employees, managers, users, suppliers, competitors, and other academic and research organizations, recently taking its most open form through network-based crowdsourcing platforms (Chen & Vanhaverbeke, 2019; Chesbrough, 2003a, b). Organizations increasingly rely on complex digital and social processes to enhance connection of internal and external organization actors to manage and support knowledge flows (Adamides & Karacapilidis, 2020; Anderson & Hardwick, 2017; Cepeda-Carrion et al., 2022; Scuotto et al., 2022). According to the organizational knowledge creation theory as developed by Nonaka and Takeuchi (1995), interaction, socialization, combination, and internalization of knowledge as well as its externalization in explicit forms are essential dimensions of the knowledge creation and innovation process. The use of digital technologies can enhance and support learning processes and build the absorptive capacity of the firm. The adoption of artificial intelligence and the possibility of decision-making based on the analysis and exploitation of big data enhance the interconnection of organizations and their capability to seize opportunities and exploit them through the innovation process. Social interactions, as well as the way information and knowledge management, integration, and exploitation are performed, can further support to a significant extent the development of organizational absorptive capacity.

Combining these long-established arguments, it is straightforward to propose the following two hypotheses:

- H2: Absorptive capacity increases a firm’s innovation performance.
- H3: Digital capacity enhances the firm’s absorptive capacity as it expands its knowledge base and supports interaction*.*

In a more detailed literature review, AC has been approached in different contexts, as a mediating factor in the process of transformation of existing or new knowledge into economic value, given that firms combine external with internal resources (Engelen et al., 2014; Rothaermel & Alexandre, 2009; Winkelbach & Walter, 2015). The discussion in this study falls within the theoretical framework that considers AC as a dynamic capability, as previously presented in this section, and more specifically we analyze AC in the context of digital transformation. We argue this presents a particular interest for the following reasons:

- Digital technologies create opportunities for all type of industries to compete, value, and manage the knowledge they hold (Carayannis et al., 2006; Rêgo et al., 2021; Scuotto et al., 2017). They can potentially transform even industries that are conventionally characterized as low-tech. The recognition of such opportunities requires a knowledge base and capability to interpret and evaluate them.
- Rapid technological evolution in this area increases the cost of implementing digital transformation but also reduces the product life cycle (Mubarak & Petraite, 2020). Thus, firms become increasingly dependent on external knowledge sources to efficiently implement digitalization (Enkel et al., 2017).
- In the digital economy era, digitalization has become more of a strategic decision rather than a technical question, as organizations integrate digital technologies to create new value and this process very often requires a reinvention of the company in terms of its business model, strategic goals, operations, and relationships with other actors (Rêgo et al., 2021; Rogers, 2016; Siachou et al., 2021; Tekic & Koroteev, 2019).

As emerging digital technologies are quickly transforming into competitive assets in the innovation activity and the pace of technological advances increases, firms need to develop their capacity to recognize opportunities deriving from the exploitation of these technologies, to assimilate and incorporate them in their strategic management, and to create new value propositions building new competitive advantages (Carayannis et al., 2006; Mubarak & Petraite, 2020; Rêgo et al., 2021; Scuotto et al., 2017; Siachou et al., 2021). The exponential change in digital technologies makes absorptive capacity a more critical element as firms need to possess a knowledge base to either follow advances and integrate them in their production and management practices or to leapfrog towards new ways of delivering value, covering the gap between what is known and what had to be established. A clear understanding of the relative advantages of digital technologies and awareness of benefits deriving from their adoption establishes a culture that nurtures and further enables the digitalization process (Alshamaila et al., 2013). Furthermore, as digital technologies apply horizontally in the service and manufacturing sector, organizations need to combine or disrupt their established routines, processes, or business models to address the undergoing challenges. In this context, learning and assimilation of external knowledge become essential in the process of the digital transformation and its successful exploitation in all aspects of the firm, which leads to the formulation of the fourth and final hypothesis of this study:

- H4: Absorptive capacity further enables the contribution of digital capacity to the firms’ innovation performance (i.e., it provides a positive mediating effect).

**Fig. 1:**
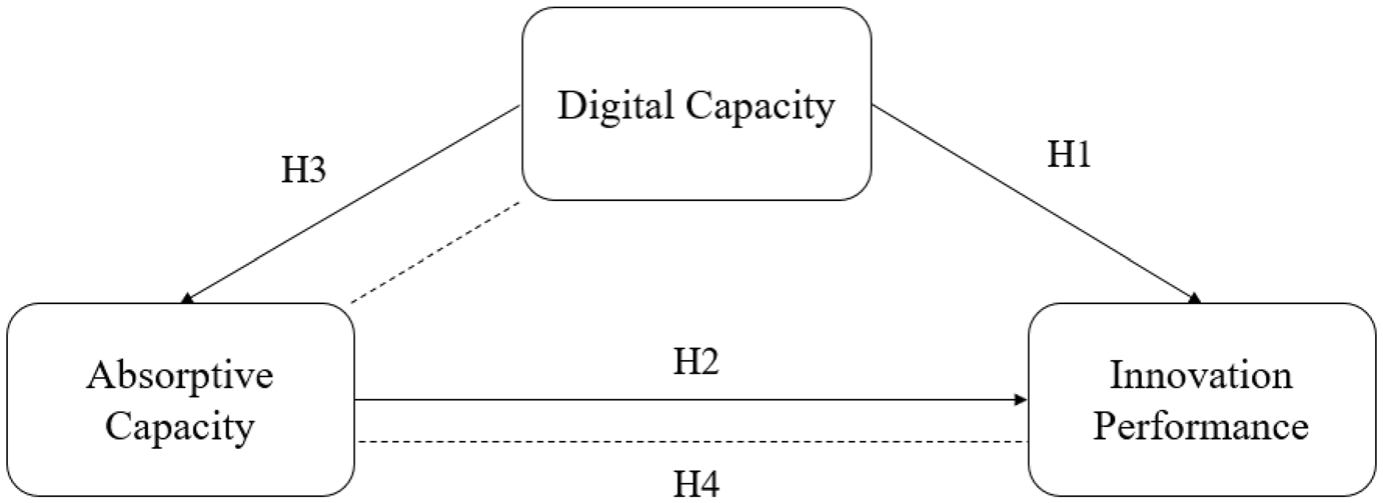
Schematic representation of the conceptual model (including the research hypotheses)

**Fig. 2:**
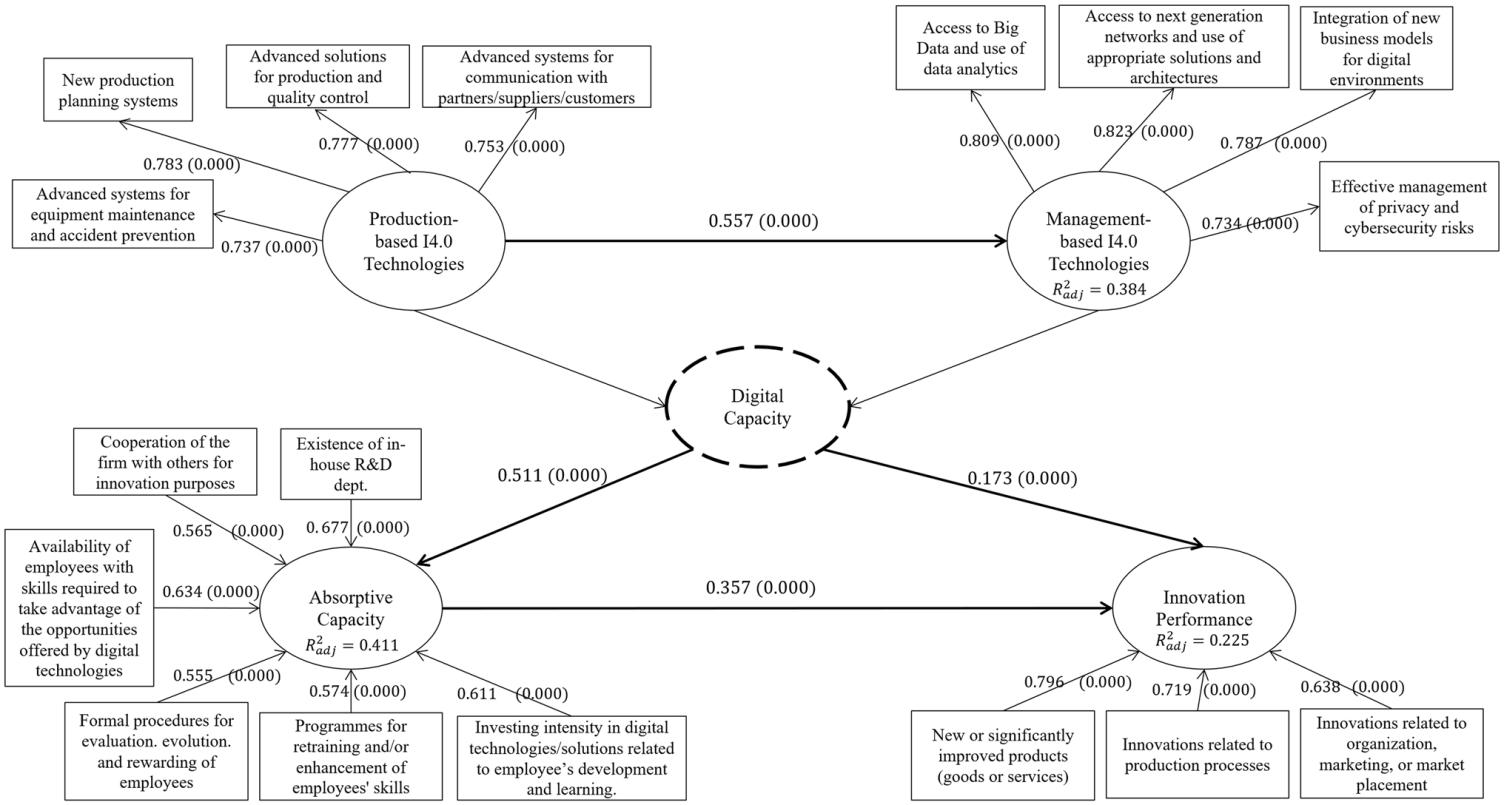
Schematic representation of the measurement and structural model with selected metrics. Notes: 1: Control variables and related links are omitted to avoid clutter; 2: for the measurement model, loadings and p-values (in parentheses) are presented. For the structural model, direct effects and p-values (in parentheses) are shown next to the corresponding link and total ${{\varvec{R}}}_{{\varvec{a}}{\varvec{d}}{\varvec{j}}}^{2}$ is shown inside the constructs. Factor loadings for DC are omitted

The arguments posed in the discussion above alongside their corresponding research hypotheses are presented in a schematic representation of our conceptual framework in Figs. 1 and 2.

## Empirical Research

Various studies have shown that productive and technological transformation in Greece was slow over the years and coupled with institutional and governance inefficiencies reflected on low and decreasing productivity and competitiveness (Caloghirou, 2008; Giannitsis, 2013; Giannitsis & Kastelli, 2014; Giannitsis et al., 2009; Kastelli & Zografakis, 2017; Papayannakis, 2008). In this context, specific structural characteristics of the Greek business sector, such as the small firm size, the relatively high contribution of labor compensation in gross production value, the low R&D investment, and the weak interactions among the actors of the business ecosystem, are major constraints of the innovation performance, as it is also depicted in the respective European Innovation Scoreboard rankings in different years. Although the Greek business sector seems to improve its innovation performance in relative terms, it presents limitations as far as supply factors are concerned: R&D intensity as well as R&D performed outwards is low and researchers in R&D are well below the EU19 and EU27 average.^2^

As far as digital transformation is concerned, Greece, although presenting some improvement, ranks low regarding relevant digitalization indicators (European Commision, 2021), and Greek SMEs still appear to lag behind the European average in assimilating new technologies and engaging in e-business activities (European Commission, 2016). Greece’s digital performance in relation to that of the EU-27 as measured with the DESI index and its sub-indicators presents an improvement in some dimensions such as connectivity and use of internet, although important issues remain to be solved, and at the same time dimensions of human capital and digital public services present a marginal improvement. A recent study on digital performance of the Greek economy by Laitsou et al. (2020) has shown that a convergence is possible in the next 10 years as a result of the development in “connectivity” and “use of internet,” but difficulties still remain in the area of “human capital” and in all indicators relating to digitization of the business sector. Furthermore, Greece ranks below the EU average of digital adopters’ labor productivity, but the critical role of digitalization can be also found in the difference between the average labor productivity of digital adopters and non-adopters, which is one of the highest in EU (European Investment Bank, 2020), indicating potential gains from digitalization. This difference also highlights the risk of a further increase in asymmetries if firms do not invest in technical, managerial, and organizational skills and intangible assets such as software and data that are required for digitalization. Greece also ranks high—23rd among 64 countries—in terms of digital trade restrictions,^3^ showing that policy responses to digitalization are sluggish and anemic.

Relevant research on Greek firms has identified the critical role of knowledge flows and absorptive capacity on innovation performance and at the same time their contribution towards the adoption of ICTs (Giotopoulos et al., 2017). In light of I4.0 revolution and faced with the new challenge resulting from the recent pandemics, Greek firms need to reshape their priorities vis-à-vis adoption, integration, and use of new technologies (Stamopoulos et al., 2022; Tsakanikas et al., 2021). The above characteristics reveal the relevance of the discussion on digital transformation and the mediating role of absorptive capacity in the Greek context. Interestingly, although firms in our research are active in terms of innovation performance, they do not undertake extensive formal R&D activities and one-third have developed innovations in cooperation with other organizations (see Table 1, column 8). Regarding digital awareness, these firms show an important lag. Only 12.3% already adopts and benefits from I4.0 technologies and 44% declared that they were either not updated or did not intend to participate in I4.0 transformation (see Table A.1 in Appendix). This indicates that either they do not have a clear understanding of the benefits arising from the adoption of digital technologies, or they are locked in activities of low value added and advantages deriving from digitalization do not concern them.

**Table 1:** Formulation and main descriptive statistics of latent constructs

**Latent var**	**Type**	**Role**	**Order**	**Description/items used**	**Type**^**1**^	**Mean**	**%**^**2**^	**N**
Production-based I4.0 technologies^3^ (PbT)	Reflective	Independent	^1st^	New production planning systems	5-Likert	2.35	24.7%	961
				Advanced solutions for production and quality control	5-Likert	2.66	32.2%	965
				Advanced systems for communication with partners/suppliers/customers (e.g., e-invoicing, digital procurement)	5-Likert	2.39	23%	960
				Advanced systems for equipment maintenance and accident prevention (e.g., sensors, predictive maintenance, devices worn for safety)	5-Likert	2.67	31.6%	958
Management-based I4.0 technologies (MbT)	Reflective	Independent	1^st^	Access to Big Data and use of data analytics	5-Likert	2.01	16.7%	941
				Access to next generation networks and use of appropriate solutions and architectures (cloud, hardware and software as a service)	5-Likert	2.49	27.4%	960
				Integration of new business models for digital environments, such as e-commerce and participative platforms	5-Likert	1.87	12.7%	954
				Effective management of privacy and cybersecurity risks	5-Likert	2.65	31.2%	951
Digital capacity (DC)	Formative	Independent	2^nd^	Production-based I4.0 technologies and management-based I4.0 technologies	-	-	-	-
Absorptive capacity (AC)	Formative	Independent/dedependent	1^st^	Availability of employees with skills required to take advantage of the opportunities offered by digital technologies	Yes/no	2.22	39.3%	982
				Formal procedures for evaluation, evolution, and rewarding of employees	Yes/no	1.46	46.2%	989
				Programs for retraining and/or enhancement of employees’ skills	Yes/no	1.64	64%	989
				Investing intensity in digital technologies/solutions related to employee’s development and learning	Yes/no	1.46	46.1%	984
				Cooperation of the firm with others for innovation purposes	Yes/no	1.33	33.4%	1004
				Existence of in-house R&D dept	Yes/no	1.27	27.1%	1008
				Number of employees with at least a bachelor’s degree *(nlog)*	Scale	1.61	-	907
Innovation performance (IP)	Formative	Dedependent	1^st^	Introduction of new or significantly improved products (goods or services) on the market during the last 2 years (2017–2018)	Yes/no	1.48	47,9%	1006
				Introduction of innovations related to production processes in the last 2 years (2017–2018)	Yes/no	1.31	30.6%	993
				Introduction of innovations related to organization, marketing, or market placement in the last 2 years (2017–2018)	Yes/no	1.26	26.4%	999
Size	**-**	Control	**-**	1.Number of full-time employees	Scale	50.6	-	956
Technological intensity	**-**	Control	**-**	1.Classification of firms on 4 categories based on their technological intensity^4^	4-point ordinal	-	26%	1004

1: Range and type of indicator, e.g., 4-point ordinal scale or 5-point Likert type scale; 2: percentage of positive answers (for yes/no items) and for the two top response categories (for 5-point Likert-type scale items); 3: an additional indicator was available from the questionnaire regarding the use of 3D printing technologies for fast prototyping/production of components but was excluded from the model as only 5.3% of the sample was using them; 4: 4 = “High-technology,” 3 = “Medium–high-technology,” 2 = “Medium–low-technology,” 1 = “Low-technology”

### Empirical Strategy and Model Specification

Our methodological approach includes the development of a composite two-stage model, which enables us to examine the multiple effects (both direct and indirect) of digital capacity on innovation while considering the mediating effects of absorptive capacity simultaneously. The two stages in the analysis include the development of the outer (or measurement) model, which describes the relationship between the indicators and the latent variables, and that of the inner (or path) model, which in turn examines the paths between the latent variables based on the underlying research hypotheses of this study. We adopt the partial least squares structural equation modeling (PLS-SEM) estimation method, which is an advanced quantitative method that can be considered as a combination of factor analysis and multiple regressions (Hair et al., 2011). Structural models typically fall under two broad categories, covariance-based models (CB-SEM), which use the covariance analysis and are considered as strict models, and variance-based models, which maximize the explained variance of the dependent variable by its explanatories (Hair et al., 2012). Our approach falls under the latter category, with a significant advantage that it can highlight the links between the latent variables and measure the effects (both direct and indirect) that they have on one another (Hair et al., 2017). Furthermore, our selection is line with the selection criteria posed by Hair et al. (2019) which, among others, suggest that PLS-SEM should be the preferable structural method when the model is complex and includes many constructs and relationships, when the inner model includes at least one formative construct, when lack of normality in the distributions is present, and when the research objective requires latent variable scores for post-estimation analysis, which are particularly relevant with our application. Furthermore, an additional motivation stems from the fact that our model includes binary variables (Hair et al., 2013). The empirical analysis is conducted with the “SmartPLS” 3.0 software (Ringle et al., 2015).

### Data Collection and Characteristics

Our research deploys data for a diverse sample of 1014 Greek firms, mainly manufacturing and from different regions and sectors, building on the results of a large field survey conducted in 2019 by the Laboratory of Industrial and Energy Economics of the National Technical University of Athens (LIEE-NTUA) in collaboration with the Greek Foundation for Economic and Industrial Research (FEIR/ΙΟΒΕ). This study aimed to map the business activity in terms of exports, innovation, global value chain participation, cooperation, and the technological transformation of the Greek business ecosystem. The survey was designed focusing on industrial ecosystems comprising manufacturing firms and a smaller number of related activities that provide support services to manufacturing.^4^ The survey was implemented through structured (closed format) questionnaires that targeted the CEOs and CFOs of the firms, utilizing the CATI method (expert-assisted interviews), achieving a response rate of 59.6% (1014 responses out of an initial target of 1700). Questionnaire results were subsequently compiled into a unified dataset, from which the current study draws data upon. More details on the sample distribution based on different criteria (size, industry classification, and technological intensity) can be found in the Appendix, in Tables A.2 and A.3. Most of the firms in the sample (Table A.3) are micro (< 10 employees) and small (11–49 employees), as they account for almost 78% of the total. Medium size firms (50–249 employees) account for 18% of the sample, and the remaining are large firms (250 and more). This structure overrepresents the number of large firms in the overall Greek population because the main goal of the survey was to select by priority the largest firms at regional level, to better capture information from firms having innovation and R&D activities while being potential users of digital technologies.^5^ Regarding technological intensity, sectors are grouped according to Eurostat and OECD R&D intensity-based classification. Sector distribution of firms within these groups is presented in Table A.2.

### Formulation of the Latent Constructs and Linkages

The first step for the development of our empirical model was the construction of latent constructs to operationalize the variables included in the conceptual model (Fig. 1), using corresponding questionnaire items which were grouped and tested using both Explanatory (EFA) and Confirmatory Factor Analysis (CFA). This procedure resulted in a set of multivariable latent constructs which were included in the empirical model (see Table 1). In detail, the set of variables includes:

*Innovation performance* (labeled as IP) – Dependent variable (formative): To measure innovation performance, we utilize information from specific questionnaire items regarding whether each firm has introduced product, process, or marketing innovation in the 2-year span preceding the survey (Yes/No binary items). While these items capture the self-perception of the respondents, they allow for a more inclusive quantification of the innovation activities of Greek manufacturing firms, as previous large-scale surveys have shown limited activity in other traditional innovation metrics such as patents/patent applications.^6^

*Product-based I4.0 technology* (labeled as PbT) – Independent variable (reflective): To develop this construct, we turn to a set of questionnaire items that capture the use of tangible I4.0 technologies that directly relate to the production process per se. This includes new production planning systems, advanced solutions for product quality control, supply chain technologies that relate to advanced systems for communication with partners/suppliers/customers, and predictive maintenance and workspace safety systems. All items correspond to Likert-scale responses, ranging from 1 to 5 depending on the use of such technologies (with 1 referring to no use and 5 to intensive use).

*Management-based I4.0 technology* (labeled as MbT) – Independent variable (reflective): For this construct, we turn to questionnaire items that capture the use of intangible, data-driven technologies that apply horizontally to all business functions and mostly relate to management. We deploy a set of 5-point Likert-scale items that reflect the use of these of technologies (same as PbT). This set includes the use of big data analytics, access to next generation network technologies (hardware, software, cloud services, etc.), integration of new digital business functions (e.g., e-commerce), and use of data protection and cybersecurity technologies.

*Digital capacity* (labeled as DC) – Independent variable (formative): To formulate a latent variable that captures digital capacity, we combine the two I4.0 technology variables (PbT and MbT), to develop a second order construct for our inner model.

*Absorptive capacity* (labeled as AC) – Independent variable (formative): We formulate this construct by turning to questionnaire items that correspond to the operationalization of absorptive capacity in relevant literature, as described in the “Absorptive Capacity as a Mediator Between Digital Capacity and Innovation Performance” section. Our set of items includes six ordinal indicators related to the availability of employees with skills required to take advantage of the opportunities offered by digital technologies; the existence of formal procedures for evaluation, evolution, and rewarding of employees; the implementation of training programs and/or enhancement of employees’ skills; the implementation of investments on digital technologies/solutions related to employee’s development and learning, whether or not the firm cooperates with others for innovation purposes; and existence of in-house R&D department. These items are further accompanied with a scale item, namely, the number of employees with at least a bachelor’s degree (taken in natural logarithm).

*Control Variables*. We further include two control variables that capture the size and the technological asymmetry effects of firms from different sectors. The former is captured through the number of full-time employees of each firm (scale), and the latter through a 4-point ordinal variable which corresponds to the OECD and Eurostat sector classification of business activities, using their corresponding NACE coding to classify them into “High-technology,” “Medium–High-technology,” “Medium–low technology,” and “Low-technology”.^7^

Details regarding the multivariable latent constructs alongside some key descriptive statistics are summarized in Table 1.

## Results

Following standard practices for assessing PLS-SEM output metrics (Hair et al., 2019), we initially evaluate the outer (measurement) model and then proceed to the inner (structural) model. While the evaluation process, its steps and interpretation of the metrics, is not absolutely standardized, there is consensus on the acceptable thresholds and relevant metrics for each step.

### Measurement Model Evaluation

The loadings of the indicators of our reflective latent variables (Table 2) are all above the typical reliability threshold of 0.7 (Hair et al., 2019), providing satisfactory item reliability with no collinearity issues emerging (the average outer VIF value is below 1.75).

**Table 2:** Loadings of the indicators on the model’s reflective latent variables

**Indicators used**	**MbT**	**PbT**	**VIF** (outer)
New production planning systems		0.783^***^	1.505
Advanced solutions for production and quality control		0.777^***^	1.576
Modern systems for communication with partners/suppliers/customers (e.g., e-invoicing, digital procurement)		0.753^***^	1.312
Advanced systems for equipment maintenance and accident prevention (e.g., sensors, predictive maintenance, devices worn for safety)		0.737^***^	1.438
Access to Big Data and use of data analytics	0.809^***^		1.597
Access to next generation networks and use of appropriate solutions and architectures (cloud, hardware, and software as a service)	0.823^***^		1.735
Adoption of new business models for digital environments, such as e-commerce and participative platforms	0.787^***^		1.565
Effective management of privacy and cybersecurity risks	0.734^***^		1.411

^***^Significant at the 0.1% level; ^**^Significant at the 1% level; ^*^Significant at the 5% level

The reliability of the internal consistency (Table 3) of our reflective latent variables is assessed using three standard metrics.^8^ In all cases, values should be higher than 0.7 and lower than 0.9 (which could indicate redundancy of some items) and our latent variables’ values fall within that acceptable range. For assessing their convergence validity, we rely on the average variance extracted (AVE) indicator, which represents the ratio of variance measured by our constructs to variance due to measurement error (Fornell & Larcker, 1981). Again, our reflective latent variables show AVE values above the suggested threshold of 0.5, indicating adequate convergence validity.

**Table 3:** Reliability metrics of the model’s reflective latent variables

**Construct**	**Cronbach’s alpha (**${{\varvec{C}}}_{\boldsymbol{\alpha }}$**)**	**Rho-alpha (**${{\varvec{\rho}}}_{\boldsymbol{\alpha }}$**)**	**Composite reliability (**${\varvec{C}}{\varvec{R}}$**)**	**Avg. variance extracted (**${\varvec{A}}{\varvec{V}}{\varvec{E}}$**)**
PbT	0.752	0.785	0.847	0.582
MbT	0.787	0.826	0.868	0.622

Single-item constructs are excluded

The assessment of the discriminant validity of the constructs is based on the heterotrait-monotrait (HTMT) ratio (Table 4) which falls within the acceptable range (below 0.85), indicating sufficient differentiation among our used constructs (Henseler et al., 2015).

**Table 4:** Heterotrait-Monotrait ratio (HTMT) of the indicators of the latent variables

**Construct**	**MbT**	**PbT**	**Size (control)**	**Technology intensity (control)**
MbT	-			
PbT	0.759	-		
Size (control)	0.132	0.121	-	
Technology classification (control)	0.304	0.143	0.009	-

The assessment of the measurement model also yields satisfying results for the formative latent variables. As it can be observed from the relevant metrics in Table 5, each formative variable presents strong and significant relationships with its respective indicators, but with the loadings of some indicators for AC falling slightly below 0.6. However, as both their loadings and the respective weights are still highly significant ($p<0.01$) and are above 0.5, they are considered satisfactory (Hair et al., 2017, 2019). Regarding the assessment of collinearity issues, all outer indicators present variance inflation factors (VIF) less than 1.45 and therefore fall well within the acceptable range (Hair et al., 2011).

**Table 5:** Relevance and significance metrics of the model’s formative constructs

**Formative constructs**	**Description/items used**	**External weight**	**External loading**	**VIF** (outer)
Absorptive capacity	Availability of employees with skills required to take advantage of the opportunities offered by digital technologies	0.243^***^	0.634^***^	1.309
	Formal procedures for evaluation, evolution, and rewarding of employees	0.201^***^	0.555^***^	1.247
	Programs for retraining and/or enhancement of employees’ skills	0.169^***^	0.574^***^	1.326
	Investing intensity in digital technologies/solutions related to employee’s development and learning	0.165^***^	0.611^***^	1.444
	Cooperation of the firm with others for innovation purposes	0.295^***^	0.565^***^	1.150
	Existence of in-house R&D dept	0.265^***^	0.677^***^	1.348
	Number of employees with at least a higher technical education *(nlog)*	0.380^***^	0.709^***^	1.221
Innovation performance	Introduction of new or significantly improved products (goods or services)	0.566^***^	0.796^***^	1.147
	Introduction of innovations related to production processes or processes	0.436^***^	0.719^***^	1.164
	Introduction of innovations related to organization, marketing, or marketing	0.389^***^	0.638^***^	1.108

^***^Significant at the 0.1% level; ^**^Significant at the 1% level; ^*^Significant at the 5% level

The strongest-loading item for AC is the number of employees with at least a bachelor’s degree, followed by whether the firms cooperate with others towards attaining innovation goals and whether they have an organized in-situ R&D department, while innovation performance appears to be formed more by the introduction of product-related innovations to the market, rather than of process or market/marketing related.

### Structural Model and Research Hypotheses Evaluation

Following the validation of the measurement model, we proceed to evaluate the relationships between the constructs (inner model) and their significance in Table 6. At first, we use the inner variance inflation factors (VIF) to check for possible collinearity issues, which yield satisfactory results for all constructs (average VIF below 2). Second, we evaluate the model’s fit on the constructs. In terms of the variance of the dependent variables explained by the model, adjusted ${R}^{2}$ stands at 0.225 ($p<0.1\%$) for innovation performance and at 0.411 ($p<0.1\%$) for AC. The MbT component of DC is also adequately predicted by PbT (${{\varvec{R}}}_{{\varvec{a}}{\varvec{d}}{\varvec{j}}}^{2}=0.384$). A schematic representation of the measurement and structural model with selected metrics is presented in Fig. 2.

**Table 6:** Quality criteria and collinearity tests of the main constructs (inner model)

**Constructs**	**R**^**2**^** adj**	**f**^**2**^** effect size**		**Inner VIF**^**1**^
		On AC	On IP	
AC	0.411^***^	-	0.097^***^	< 1.704
DC	-	0.415^***^	0.026^*^	< 1.517
PbT	0.027^***^	-	-	< 1.529
IP	0.225^***^	-	-	< 1.791
MbT	0.384^***^	-	-	< 1.528
Size (control)	-	0.069^**^	0.001	< 1.086
Tech. intensity (control)	-	0.062^***^	0.002	< 1.121

R^2^ adjusted of digital capacity omitted as it is measured via repeated indicators by its components

^***^Significant at the 0.1% level; ^**^Significant at the 1% level; ^*^Significant at the 5% level

DC presents a significant effect size on AC ($${f}^{2}=0.415, p<0.01)$$, while AC’s effect size on IP is smaller ($${f}^{2}=0.097, p<0.1\%)$$ but highly significant as well. Interestingly, DC has the smallest and less significant effect size on IP ($${f}^{2}=0.026, p<5\%)$$. Our control variables show minor effect sizes on the firm’s AC ($${f}^{2}=0.069, p<5\%$$ for size and $${f}^{2}=0.062, p<0.1\%$$) for technological intensity, with their significance levels being very different. Both control variables do not appear to have any significant or sizable effect on IP.

The inner (path) model results are presented in Table 7. In detail, the relationships between DC, AC, and IP present the expected positive direction and effects in each case and align with our corresponding research hypotheses (H1–H3). The strongest direct effect is observed between DC and AC ($\beta =0.511, p<0.1\%)$, followed by AC’s direct effect on IP ($\beta =0.357, p<0.1\%)$ and DC’s effect on IP ($\beta =0.173, p<0.1\%)$, respectively. Our control variables differ in terms of effect strength and significance on our dependent variables. Technological intensity positively affects AC ($\beta =0.196)$, PbT (*β* = 0.131), and MbT (*β* = 0.195) at the 0.1% significance level. This finding is not surprising as more technological intensive firms are expected to invest more in R&D, training, and other activities that develop their absorptive capacity. A similar explanation goes for size, which has stronger and more significant effects ($p<0.1\%$) on AC ($\beta =0.203$) and on PbT ($\beta =0.106$), respectively, and to a lesser extent to MbT ($\beta =0.053) \mathrm{at }5\mathrm{\% level of significance}$.^9^

**Table 7:** Direct and indirect effects among constructs, and testing of hypotheses

	**IP**		**AC**		**PbT**		**Mbt**	
	**Direct**	**Indirect**	**Direct**	**Indirect**	**Direct**	**Indirect**	**Direct**	**Indirect**
Size (control)	0.017	0.116^***^	0.203^***^	0.063^**^	0.106^**^	-	0.053^*^	0.059^**^
Tech intensity (control)	− 0.018	0.151^***^	0.169^***^	0.117^***^	0.131^***^	-	0.195^***^	0.073^*^
AC	0.357^***^ (H2)	-	-	-	-	-	-	-
DC	0.173^***^ (H1)	0.182^***^ (H4)	0.511^***^ (H3)	-	-	-		-

The positive direction is from row item to column item. Constructs which are not linked do not correspond to an effect type and are omitted

^***^Significant at the 0.1% level; ^**^Significant at the 1% level; ^*^Significant at the 5% level

It also becomes apparent that DC and size also affect innovation performance indirectly in a significant manner through AC, which partially mediates the first relationship (DC-IP) and fully the second (size-IP). Specifically, the indirect effect of DC on IP through AC ($\widehat{\beta }=0.182, p<0.1\%)$ is stronger than the direct effect of DC to IP ($\widehat{\beta }=0.173, p<0.1\%)$, supporting our fourth research hypothesis (H4). Furthermore, this difference in effect strength between the direct and indirect interaction of DC on IP was found to be significant at the $a=5\%$ level when testing it through a 10,000-sample bootstrapping parameter estimation $(0.0091>\widehat{\beta }-\beta >0.0116, p<5\%)$. In summary, our empirical results along with the corresponding research hypotheses are presented in Table 7.

## Discussion

Reiterating the main points of the presentation of our empirical findings in the section above, our results support the hypotheses proposed in the theoretical framework and can be summarized in the following four key points:

- Digital capacity presents both direct and indirect positive effects on innovation performance (H1).
- The indirect effect of DC, mediated by absorptive capacity, is stronger than the direct effect (H4).
- Digital capacity has a strong and positive direct effect on absorptive capacity as it expands its knowledge base (H3).
- Absorptive capacity presents a strong and significant contribution to innovation performance (H2).

As discussed in our conceptual framework, a number of studies have highlighted the mediating role of absorptive capacity in leveraging adoption of digital technologies and the fact that the digital capacity is not an unquestionable asset for innovation performance. Our findings align with this discussion as they unveil a complementarity of digital and absorptive capacity as enablers of innovation. Both present a positive direct contribution to innovation performance with the DC direct effect being weaker. Digital capacity presents a stronger effect on innovation performance when mediated by the effect of absorptive capacity. Hence, the mere adoption and use of digital technologies are not sufficient boosters of innovation. These results corroborate the findings of Usai et al. (2021), Scuotto et al. (2022), and Siachou et al. (2021) that put forward the complex interdependencies when targeting digital transformation.

Another set of results is also important in linking specific aspects of digital technologies to innovation performance. We measure digital capacity through the adoption and use of production-based and management-based technologies, and we find a combined effect on innovation performance. The link between ΡbΤ and ΜbΤ points to the importance for firms to formulate their digitalization strategy integrating both physical technologies related to production processes and innovation development per se, with intangible technologies that support the overall business functions and the innovation process indirectly. There is need for a holistic approach in the adoption of DTs. Technology counts as a physical asset but also intangible aspects ensure the integration of emerging technologies in the overall digital capacity building and innovation performance.

Additionally, some interesting results emerge on the relationship between the size and the type of I4.0 technologies (production and management-based). The positive and highly significant direct effect of size on PbT could very well be linked to the issue of economies of scale, that is, larger companies extensively use this type of technologies as they can secure higher returns faster. While we cannot argue on the unilaterality of the direction of this relationship, we can assume that it can work both ways: larger firms are in a better position to adopt and exploit DTs but also the adoption of DTs can work as a booster for increasing their market share and thus size (either through diversification or through productivity increase).

As it appears in the context of the Greek manufacturing firms, given their low performance in different innovation and digitalization indicators, it is not straightforward that an overall industrial strategy boosting digitalization will result to the required improvement of innovation performance in the Greek business ecosystem.

Besides the general assumption that digitalization amplifies innovation, the direct although moderate effect of digital capacity to innovation performance might relate also to the specific sample. Greek firms lag considerably behind in the adoption of I4.0 technologies and thus any improvement in that respect can more explicitly result into innovation gains than in more technologically advanced contexts, a fact that could be related to a catch-up effect. However, even though this can be valid, the mediating role of AC points to the fact that such technologies cannot stand alone as sources of competitive advantage. Other elements interfere and their absence could jeopardize the process of digitalization and the positive effects from digital transformation. Internal R&D and interactive efforts to expand the firm’s knowledge base and innovation capabilities are critical contributors to their innovation performance and can enhance returns on investment to highly sophisticated technologies. As we approach absorptive capacity looking at its specific constituent elements, our results point to specific factors that could play a critical role in digital transformation such as training and R&D efforts, educational level of employees, and cooperative efforts. In this context, policy aiming to boost digitalization of Greek firms should consider the need for improvement in other aspects of the business ecosystem that relate to innovation, such as R&D efforts, training, interaction among actors, and building of communities of practice to efficiently address the challenge of digital transformation. Policy design and implementation should take into consideration the diversity in firms’ capabilities and foster the upgrading process not only in the adoption of digital technologies but also of their capability to integrate and assimilate new knowledge and transform it into different types of innovation outcomes.

Moreover, as digital technologies cut across the knowledge base of different industrial sectors, the digital awareness of Greek firms in different sectors is mediated by their capability to integrate and assimilate new knowledge and transform it into different types of innovation outcomes. This is particularly relevant for lower technology sectors, as the greater technological lag they present can also constitute a clearer margin for technological upgrade that can then considerably increase their productivity through the adoption of I4.0 technologies.

Finally, it should be considered that as several studies provide evidence on a path dependence in adoption of new technologies based on previous innovation experience and knowledge of specific technologies, the development of firms’ absorptive capacity can establish a virtuous cycle of ICT adoption–innovation–further exploitation of opportunities deriving from ICT development, notwithstanding the support of an early adoption strategy.

## Conclusions

This paper builds on previous literature that puts emphasis on the role of digital emerging technologies in accelerating innovation as they create opportunities to generate new value through digitally driven business models, radically new products, transformation of the firm’s knowledge base, creation of new solutions to existing needs, and the restructuring of business functions.

Innovation activity is a complex process relying among other things on prior knowledge and intensity of efforts or commitment in problem solving and on interaction with external sources of knowledge. As presented in our theoretical discussion, the mediating effect of absorptive capacity on innovation has been extensively studied in the literature. On the other hand, when coming to the discussion on enablers and constraints of digital transformation, a few studies show that adopting digital technologies does not necessarily result directly to innovation.

We expand this discussion and study the contribution of digital capacity on innovation performance, proposing the mediating role of absorptive capacity in the context of digital transformation. Our results contribute to the better understanding of the underlying mechanisms through which firms can leverage their digital capacity to accelerate innovation. The empirical analysis concerns the specific case of the Greek industry. Our results highlight the strong positive direct contribution of absorptive capacity and to a lesser extent of digital capacity to innovation performance. They also support the important mediating role of absorptive capacity in enhancing the positive effects of digitalization. It is then highlighted that digital capacity is not an unquestionable asset for innovation performance.

Our results have important policy implications, as boosting the digitalization of Greek firms should consider the need for improvement in other aspects of the business ecosystem that relate to innovation, such as R&D efforts, training, interaction among actors, and building of communities of practice to efficiently address the challenge of digital transformation. Policy design and implementation should take into consideration the diversity in firms’ capabilities and foster the upgrading process not only in the adoption of digital technologies but also of their capability to integrate and assimilate new knowledge and transform it into different types of innovation outcomes.

## Limitations and Suggestions for Future Research

This paper provides motivation for future research that should aim to extend the framework and tackle certain limitations. First, the direct effect of digital capacity on innovation performance might differ according to the type of innovation. Future efforts should investigate the extent of this effect on product, process, marketing, or business model innovation separately.

Furthermore, the measurement of factors that correspond to complex constructs encompasses many challenges. Our approach of absorptive and digital capacity is grounded on extensive research on the definition and operationalization of both terms, as discussed in the “Conceptual Framework” section. However, some aspects remain unobserved. One relevant example relates to the static nature of the study, which uses data for 2019. Several aspects of the underlying phenomena that this study touches upon are rather dynamic in nature and future efforts should aim to integrate this dimension in the analysis (a relevant example is the “knowledge stock” aspect of absorptive capacity).

Our research operationalizes the formulated conceptual framework for the case of Greek firms. It is important to study the interplay of digital and absorptive capacity for innovation performance in other national contexts to generalize the results obtained and reinforce the arguments posed by this paper. Another line of possible future research revolves around the interplay of digital and absorptive capacity and its impact on different performance measures, such as sales and exports, to capture other dynamic effects of digital transformation.

Finally, in depth analysis using case studies could shed light on the way specific characteristics and established routines of the firm that relate to knowledge accumulation and creation leverage digitalization.

## Appendix

**Table A.1** Digital awareness of Greek manufacturing firms

**Table:**

**Q: To what extent does your firm monitor and participate to the I4.0 industrial revolution?**	**Frequency**	**Percentage**
Is not up to date	323	31.9
Is not up-to-date and does not intend to participate	93	9.2
Is up-to-date and intends to participate but has yet to develop a relevant strategy	344	33.9
Has developed a relevant strategy but has not yet invested	43	4.2
Has invested but is not yet making productive use of the relevant technologies	29	2.9
Is already utilizing and benefiting from I4.0 technologies	117	11.5
**Total valid**	949	93.6
**Missing**	65	6.4
**Total**	1014	100.0

**Table A.2** Composition of the sample by NACE rev1.1 industry classification and technology intensity*

**Table:**

**Tech classification**	**NACE 1.1**	**Label**	**Frequency**	**Percentage**
**High tech (15.14%)**	22	Publishing, printing, and reproduction of recorded media	57	5.68%
	24	Manufacture of chemicals and chemical products	12	1.20%
	33	Manufacture of medical, precision and optical instruments, watches, and clocks	12	1.20%
	35	Manufacture of other transport equipment	1	0.10%
	72	Computer and related activities	70	6.97%
**Medium–high tech (10.86%)**	24	Manufacture of chemicals and chemical products	37	3.69%
	29	Manufacture of machinery and equipment n.e.c	33	3.29%
	31	Manufacture of electrical machinery and apparatus n.e.c	16	1.59%
	32	Manufacture of radio, television, and communication equipment and apparatus	5	0.50%
	34	Manufacture of motor vehicles, trailers, and semi-trailers	3	0.30%
	35	Manufacture of other transport equipment	15	1.49%
**Medium–low tech (21.41%)**	13	Mining of metal ores	1	0.10%
	23	Manufacture of coke, refined petroleum products, and nuclear fuel	5	0.50%
	25	Manufacture of rubber and plastic products	50	4.98%
	26	Manufacture of other non-metallic mineral products	72	7.17%
	27	Manufacture of basic metals	11	1.10%
	28	Manufacture of fabricated metal products, except machinery and equipment	76	7.57%
**Low tech (52.59%)**	14	Other mining and quarrying	17	1.69%
	15	Manufacture of food products and beverages	248	24.70%
	17	Manufacture of textiles	34	3.39%
	18	Manufacture of wearing apparel; dressing and dyeing of fur	37	3.69%
	19	Tanning and dressing of leather; manufacture of luggage, handbags, saddlery, harness, and footwear	4	0.40%
	20	Manufacture of wood and of products of wood and cork, except furniture; manufacture of articles of straw and plaiting materials	18	1.79%
	21	Manufacture of pulp, paper, and paper products	24	2.39%
	36	Manufacture of furniture; manufacturing n.e.c	43	4.28%
	37	Recycling	2	0.20%
	40	Electricity, gas, steam, and hot water supply	38	3.78%
	45	Construction	3	0.30%
	50	Sale, maintenance, and repair of motor vehicles and motorcycles; retail sale of automotive fuel	6	0.60%
	51	Wholesale trade and commission trade, except of motor vehicles and motorcycles	2	0.20%
	52	Retail trade, except of motor vehicles and motorcycles; repair of personal and household goods	1	0.10%
	63	Supporting and auxiliary transport activities; activities of travel agencies	38	3.78%
	74	Other business activities	2	0.20%
	90	Sewage and refuse disposal, sanitation and similar activities	11	1.10%
		**Total**	**1004**	**100%**

*Technology intensity follows the Eurostat and OECD sector classification according to sectors’ R&D intensity. We grouped high tech knowledge intensive services under the high tech category, whereas other less intensive services to manufacturing are grouped under the low tech category

Table A.3 Composition of the sample by firm size classification

**Table:**

	**Frequency**	**Percentage**
1–10 employees (micro-enterprises)	273	26.9
11–49 employees (small enterprises)	514	50.7
50–249 employees (medium enterprises)	181	17.9
> 250 employees (large enterprises)	28	2.8
Total	996	98.2
**Missing**	18	1.8
**Total**	1014	100.0

## Footnotes

1 See for example the latest DESI rankings (European Commision, 2021).

2 According to the most recent structural data from Eurostat.

3 Based on the Digital Trade Restrictiveness Index (DTRI), which measures how 64 countries in the world restrict digital trade. It is argued that restrictions drive up costs for businesses as well as for consumers, whereas free digital trade provides consumers with better access to services and goods and helps businesses in all sectors of the economy to become more efficient and reach new customers (ECIPE, 2018).

4 Based on NACE revised 1.1 classification.

5 Sole proprietorship firms were detracted as it has been assumed that they are not relevant to address the topics of interest studied in the field research.

6 This is a persistent finding for Greece in the relevant rankings of the European Innovation Scoreboard.

7 OECD’s and Eurostat’s sector classification is based on the share of R&D expenditure to value added to classify 2-digit and 3-digit manufacturing activities into the four categories referenced in the text.

8 The first metric, Cronbach’s alpha (${C}_{\alpha }$), is generally thought to be a more conservative reliability estimate. The second metric, composite reliability ($CR$) (Jöreskog, 1973), is usually seen a possible over-estimator, while the third metric, Dijkstra & Henseler (2015)’s Rho-alpha (${\rho }_{\alpha }$), can provide a “middle-ground” and more accurate estimation of a construct’s reliability.

9 Of course, this can be partially attributed to the survey design to some extent.
